# A Bibliometric Analysis on Research Regarding Residential Segregation and Health Based on CiteSpace

**DOI:** 10.3390/ijerph191610069

**Published:** 2022-08-15

**Authors:** Yanrong Qiu, Kaihuai Liao, Yanting Zou, Gengzhi Huang

**Affiliations:** 1 School of Architecture and Urban Planning, Guangdong University of Technology, Guangzhou 510060, China; 2 School of Geography and Planning, Sun Yat-sen University, Guangzhou 510275, China

**Keywords:** residential segregation, health, disparity, CiteSpace, research hotspot, research frontier

## Abstract

Considerable scholarly attention has been directed to the adverse health effects caused by residential segregation. We aimed to visualize the state-of-the-art residential segregation and health research to provide a reference for follow-up studies. Employing the CiteSpace software, we uncovered popular themes, research hotspots, and frontiers based on an analysis of 1211 English-language publications, including articles and reviews retrieved from the Web of Science Core Collection database from 1998 to 2022. The results revealed: (1) The Social Science & Medicine journal has published the most studies. Roland J. Thorpe, Thomas A. LaVeist, Darrell J. Gaskin, David R. Williams, and others are the leading scholars in residential segregation and health research. The University of Michigan, Columbia University, Harvard University, the Johns Hopkins School of Public Health, and the University of North Carolina play the most important role in current research. The U.S. is the main publishing country with significant academic influence. (2) Structural racism, COVID-19, mortality, multilevel modelling, and environmental justice are the top five topic clusters. (3) The research frontier of residential segregation and health has significantly shifted from focusing on community, poverty, infant mortality, and social class to residential environmental exposure, structural racism, and health care. We recommend strengthening comparative research on the health-related effects of residential segregation on minority groups in different socio-economic and cultural contexts.

## 1. Introduction

Health is an eternal topic of human concern. With the evolution of cities and the spread of urbanization, lifestyle changes have caused new health problems [1], such as poor mental health and the rising prevalence of many chronic diseases [2]. Although chronic diseases are associated with genetic and individual differences, a growing amount of research shows a strong relationship between illness and the environment in which one lives [3]. Among them, the adverse health outcomes and inequality caused by residential segregation have received extensive academic attention [4,5].

As early as 1950, Dr. Alfred Yankauer noticed that Black infant mortality rates rose with the increase in the concentration of the Black population [6]. This marked the first time that residential segregation was linked to an adverse health outcome. However, it was only in the late 1980s and early 1990s that researchers started to investigate how residential segregation affects minority–majority group health disparities [7,8,9,10,11]. From then on, residential segregation and health (RSH) became an important research topic.

Residential segregation refers to the degree to which two or more social groups live apart in different sections of a city or metropolitan statistical area [12]. Segregation is believed to be the root cause of racial health disparities [13]. Moreover, the structural racism associated with segregation is equally critical in affecting minority health; it promotes racial discrimination through various systems such as housing, employment, income, health care, and credit, and reinforces pre-existing patterns of inequality. Most of the time, segregation promotes the concentration of poverty; it leads to a reduction in available resources such as health care [14,15,16], healthy food shops [17,18,19], and green spaces [20,21], in some cases along with air pollution exposure [22,23], all of which are significant reasons for causing and keeping minority or low-income populations in adverse health conditions [24,25,26,27].

Many studies have demonstrated that residential segregation affects the health outcomes of minority racial groups, resulting in many negative health outcomes. For example, the relationship between adverse birth outcomes and segregation among pregnant women varies by race in the U.S. with Black mothers facing a greater risk of pre-term births and low birth weight than White mothers [28]. Hypertension prevalence is higher among non-Hispanic Black (57.1%) than non-Hispanic White (43.6%) adults [29]. Residential segregation has also led to disparities in transmitted diseases, with higher infection rates of chlamydia among isolated Blacks than isolated Whites in U.S. counties that are both integrated and majority Black, respectively [30]. In the context of the global COVID-19 outbreak, studies suggest that segregated Black minorities have higher rates of infection than Whites [31,32,33]. However, minority groups have their own privileges or advantages related to living in a segregated environment. For instance, compared with majority groups, the average body mass index [34], coronary heart disease mortality rate [35], and diagnosis of late-stage colorectal cancer [36] are lower among Blacks.

Some studies indicate that living in minority areas, also known as ethnic enclaves, can provide a protective effect for health. This protective effect is also called the racial density or enclave effect [37,38]. For example, Hispanic-concentrated communities are associated with lower odds of hypertension and asthma among Hispanics [39,40], and the racial segregation of Asians is weakly and negatively associated with COVID-19 incidence, as well as lower odds of an advanced breast cancer diagnosis [41,42]. All these findings underline the health-promoting role of ethnic enclaves. However, related findings also imply that racial density is likely to promote health only when group size reaches a certain density and there is a geographically concentrated settlement pattern [43].

In this paper, we used the definition of health developed by the World Health Organization (WHO): “health is a state of complete physical, mental and social well-being and not merely the absence of disease or infirmity” [44]. Most studied health outcomes related to residential segregation, including physical, maternal, child, self-rated, and mental health, are determined by a combination of individual factors (e.g., race, gender, education level, and economic conditions), environmental conditions (the built environment, one’s social environment), and background conditions (e.g., the policy system and racism) at multiple levels [45,46].

This article focuses on the health effects caused by residential segregation using CiteSpace 6.1.R2 (64-bit) software to analyze relevant English literature retrieved from the Web of Science from 1998 to 2022. Bibliometric analysis can help us to quickly identify the development context, research hotspots, and frontiers of the academic field through graphical visualization, and to explore content that needs to be improved. In turn, this study contributes to existing research in three ways. First, compared with past literature reviews [4,47,48,49], this is the first study to our knowledge to employ CiteSpace to conduct a systematic, bibliometric analysis of RSH research. It will provide new insights not comprehensively explored in previous studies. Second, this study identifies the influential publications, countries, core researchers, and institutions as well as relationships between them. This will allow scholars to understand the research situation and content of RSH more intuitively. Finally, the most important contribution is the list of key research trends over time, which will provide a reference for follow-up research, help to strengthen our awareness, and enrich disciplinary knowledge. Therefore, we focus on the following research questions (RQs).

RQ1: Who are the major contributors (journals, authors, institutions, and countries) to the field of RSH?

RQ2: What are the main research topics and hotspots for RSH research?

RQ3: What are the frontiers and emerging trends in the field of RSH?

This study consists of the following sections: We first introduce the materials and methodology (Section 2), and then explore the temporal distribution of publications and journals and the primary contributors to the field of RSH (Section 3 and Section 4). Third, we discuss landmark literature and research topics (Section 5), followed by analysis of research hotspots and frontiers (Section 6). Finally, we state our conclusions and outlook for future research (Section 7 and Section 8).

## 2. Materials and Methodology

### 2.1. Data Sources

We derived the literature under study from the core collections (SSCI, SCI-expanded, and A&HCI) of the Web of Science (WOS) database. On 5 August 2022, we retrieved a total of 1294 articles using the following search terms with associated meanings: topic = residential segregation and health; language = English; year = all; document type = articles and reviews. The earliest collection of literature on RSH retrieved from the WOS core database dates back to 1976. Thus, we selected literature published between 1976 and the present for analysis.

### 2.2. Research Method

For literature-based research it is important to understand the field in question, hotspots, and trends. With the rise of data, the mining of research hotspots, as well as trends in the existing literature based on the scientific method, have become widespread. In this context, CiteSpace, as a visualization software for bibliometrics, has been broadly used for literature-based research and the writing of reviews [50,51]. Studies have been conducted using CiteSpace to provide a panoramic perspective on the development of various health disciplines [52,53]. The software identifies the central knowledge domain, key research topics, and development trends in a certain field through visual knowledge maps grounded in keyword co-occurrence, document co-citation clustering, and burst detection [54,55]. The nodes and connections are critical structures for visualization. The larger the node, the more frequent and centrally it occurs. The connections represent the lines between nodes. The number and thickness of connected paths describe how closely objects are connected. In addition, CiteSpace can identify nodes that manifest high-frequency changes in a short period by executing burst detection. Based on this application, we can judge the declining or rising status of a topic and whether it has reached the research frontier [56].

We performed a comprehensive bibliometric analysis of the retrieved literature on RSH using CiteSpace 6.1.R2 (64-bit). We first analyzed authors/institutions/country collaborations with significant contributions, then examined highly cited studies and identified clusters with co-citations. Next, we identified keyword co-occurrences and clusters, and finally, we detected emerging keywords. We later combined the findings of the bibliometric analysis and our review of key literature to sort out trends in RSH research and to provide meaningful guidance for future disciplinary development. We set the parameters in CiteSpace as follows: (1) timespan = 1976–2022; (2) year per slice = 1; (3) node type = author/institution/country/keyword/reference; (4) threshold selection criteria = the top 25 results for each time slice. We set the other parameters by default.

## 3. The Temporal Distribution of Publications and Journals of RSH Research 

**Figure 1:**
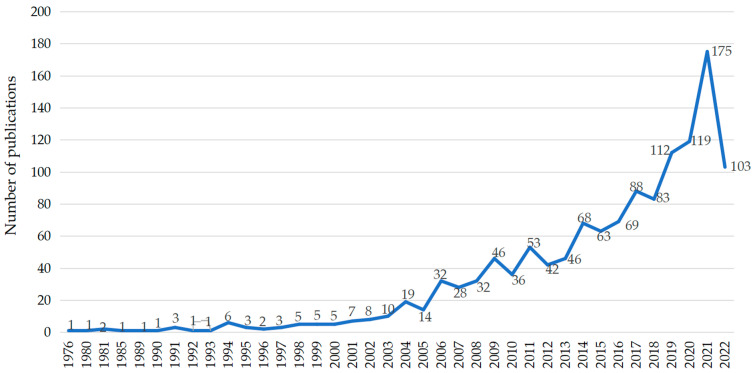
The annual number of RSH-related studies published from 1976 to 2022.

Figure 1 depicts 1294 publications from 1976 to 2022 with an overall fluctuating upward trend in the number of studies. Scholars’ attention to RSH research has gradually increased. The lowest outputs appeared before 1993 with only one or two papers published annually. The peak occurs in 2021, with the number of publications reaching 175. One possible explanation is that the negative health effects of residential segregation have been amplified by the COVID-19 pandemic, particularly the differences in COVID-19 infection and mortality rates by race [57,58]. An interesting finding is that the total number of papers published in the past 6 years is greater than the cumulative total published between 1976 and 2016. According to the papers we identified, we divided the research progress of RSH into the following three stages:

(1) Initial stage (before 2003): A fairly small number of papers was published in this period, with an average of fewer than 10 papers per year. From 1976 to 2003, only 66 papers were included, accounting for 5.1% of all publications;

(2) Development stage (2004–2013): This stage showed a fluctuating upward trend; 348 papers were published, indicating that the research on RSH had drawn scholars’ attention. However, the degree of research was not high, and the average annual number of publications did not exceed 60;

(3) Rapid development stage (2014–2022): Starting in 2014, the growth trend of the number of published papers began to accelerate. This figure rose every year except for a slight decline in 2015 and 2018, which indicates that RSH research started to become an important topic in 2014.

Table 1 shows the 10 most popular journals in RSH research. The number of publications from the top 10 journals accounts for 30.60% of all papers. Social Science & Medicine ranked first among 97 publications (7.50%), making the most positive contributions to RSH research, followed by the Journal of Urban Health–Bulletin of the New York Academy of Medicine, Health & Place, and the International Journal of Environmental Research and Public Health. The top 10 journals focus on medicine, public health, racial health, and epidemiology, reflecting the close links among sub-topics in the field of RSH.

## 4. Major Contributors to RSH Research

Co-authorship analysis examines the central forces in the research field (i.e., the main authors, institutions, and countries) and helps us to understand the close cooperation among different authors, institutions, and countries. In the network map of co-authorship, the nodes represent authors/institutions/countries, the size of a node reflects the number of published papers, and the thickness and number of lines reflect the degree of collaboration and closeness.

### 4.1. Co-Author Analysis

**Figure 2:**
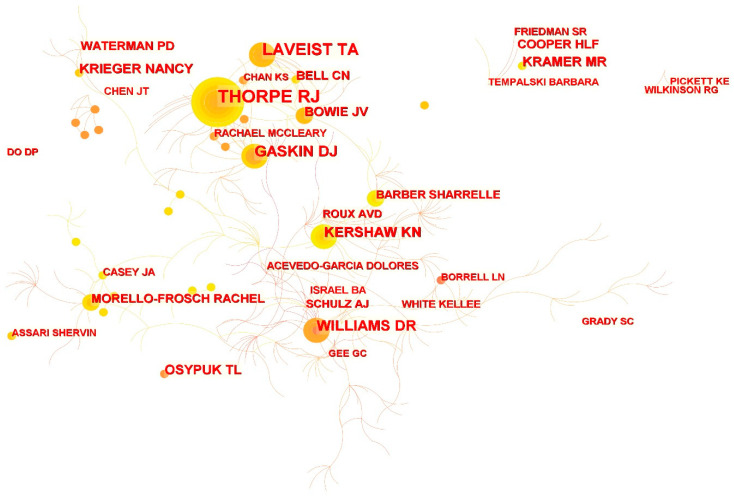
Network map showing authors’ collaborations in RSH research.

Using CiteSpace to analyze author collaboration networks in the literature, we explored the core authors in the field and the closeness of their joint efforts. Table 2 lists the top 10 authors in RSH research, with Thorpe making a significant contribution to the field with 38 articles, followed by LaVeist (29), Williams (20), Gaskin (19), and Kershaw (16). The top 10 authors are the leading scholars in the field of RSH research. The collaboration between authors is further outlined in Figure 2, which contains 1798 nodes and 1703 connections, with a network density of 0.0011. Although the network density reveals less cooperation among scholars, some nodes can be seen intuitively in the figure, indicating that the core authors in RSH research have emerged, and some studies involved teamwork. For example, the interests of a team of scholars centered on Thorpe, LaVeist, and Gaskin include exploring disparities in health outcomes [59,60,61,62,63] and health care utilization [64,65] among different races by combining factors such as race, place, and socio-economic background.

### 4.2. Co-Institution Analysis

**Figure 3:**
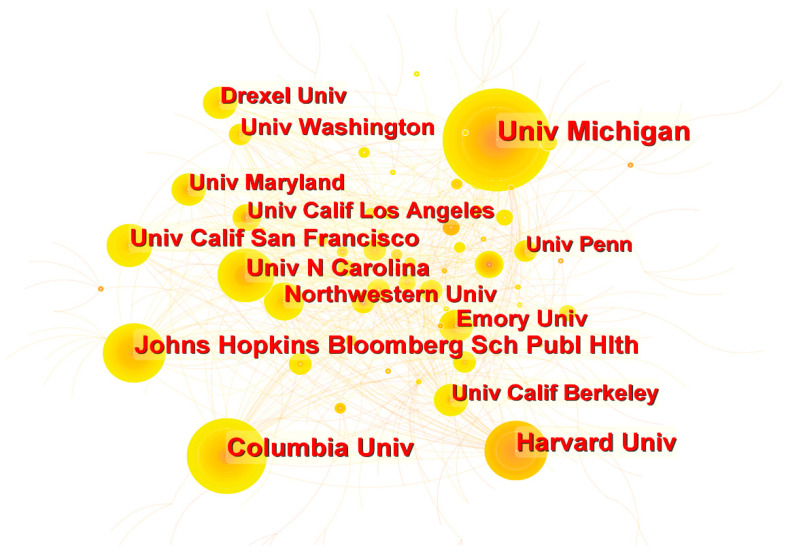
A network map showing institutional collaborations in RSH research.

The network map in Figure 3 provides information on institutional collaboration in the field of RSH. The figure contains 443 nodes and 702 connections with a network density of 0.0072. A total of 384 institutions were involved in the field of RSH from 1976 to 2022. Figure 3 portrays cooperative ties among various institutions, forming a core group of institutions comprising well-known universities such as the University of Michigan, Columbia University, Harvard University, the Johns Hopkins School of Public Health, and the University of North Carolina. An interesting finding is that the top 10 institutions are all universities (Table 3), indicating that universities form an important research force in RSH. The University of Michigan ranked first with 95 publications and was one of the first universities to research RSH. Other institutions with high numbers of publications include Columbia University (68), Harvard University (59), the Johns Hopkins School of Public Health (55), and the University of North Carolina (51).

### 4.3. Co-Country Analysis

**Figure 4:**
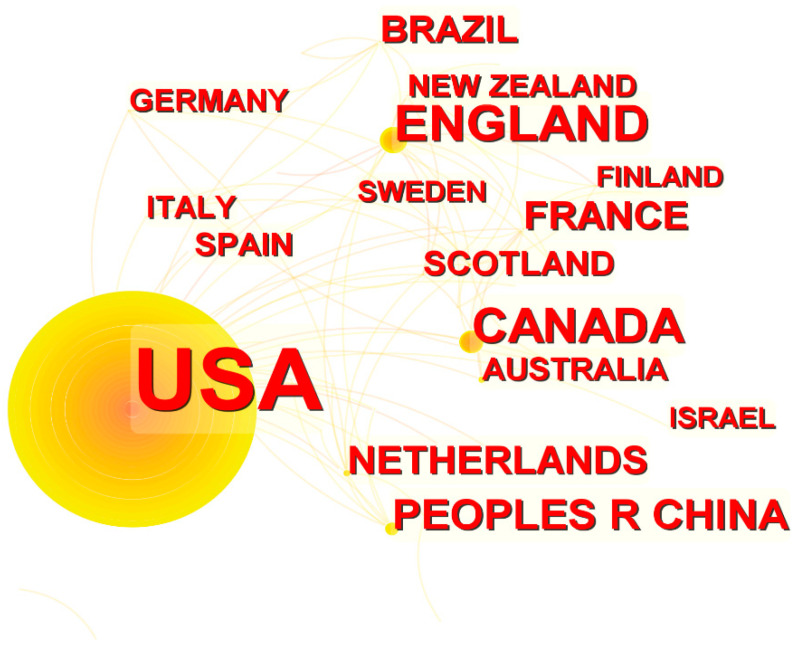
A network map showing national collaborations in RSH research.

Using CiteSpace to analyze the number of articles published in different countries, we directly identified the countries in which RSH is a popular topic (Figure 4). The network map shows that the top 10 countries, in terms of the number of publications, are all developed Western nations except for the People’s Republic of China (PRC) and Brazil. This signals that Western countries pay closer attention to RSH research. Among them, the U.S. is the leading contributor with 1107 publications (85.55%), followed by England (3.71%), Canada (3.10%), and the PRC (1.85%). The health inequalities caused by residential segregation are prominent in these countries and have received much attention from scholars and the public, resulting in more articles being published and perhaps explaining why some countries have dominated RSH research while others have little research regarding RSH. This does not mean that the problem of health inequality in other countries is not prominent, but it is very likely that scholars and institutions in other countries have not paid enough attention to it.

## 5. Landmark Literature and Research Topic Identification

### 5.1. Landmark Literature: Co-Citation Analysis

The number of citations is an important index for gauging academic value and reflects an academic circle’s recognition of findings. The analysis and interpretation of quality literature can help us to identify popular topics in the field of RSH. Using CiteSpace, we used co-citation analysis to identify landmark studies with high co-citation frequency; 13 reviews, 26 quantitative empirical studies, 10 qualitative empirical studies, and 1 book were included in the top 50 MCPs. Osypuk was included with five articles followed by Gaskin (3), Williams (3), and Kershaw (3). Exploring the health effects of residential segregation from different perspectives is a constant concern for scholars, including racism, neighborhood deprivation, neighborhood socioeconomic and built environment, and racial density [48,66,67,68,69,70]. Health outcomes are primarily related to maternal and infant health (e.g., low birth weight and preterm births) [71,72,73] and chronic diseases (e.g., obesity and diabetes) [69,74]. In terms of infectious diseases, only COVID-19 is highlighted [31]. Participants are mostly Black and White followed by Hispanics [75,76,77].

Table 4 portrays the top 10 references in RSH research; 3 papers were co-cited more than 40 times, and all papers were co-cited more than 30 times. Regarding content, 3 reviews, 6 empirical studies (including quantitative and qualitative ones), and 1 book were included. Three papers systematically review studies on racially based RSH and propose new directions for future research, such as enhancing the use of longitudinal data, adopting accurate segregation measures, and developing better pathway frameworks [4,45]. Two papers provide evidence for the link between racism and health inequality, exploring how racism systematically affects health inequality through various initiatives [48,49,78]. The study by White et al. identified racial residential segregation as a fundamental contributor to health disparities and an important factor in place-based health care inequities [13,79].

Furthermore, two empirical studies involving multilevel analysis demonstrated an association between racial residential segregation and health outcomes. A high degree of isolation was associated with poorer self-rated health among Blacks [76], and residential segregation and neighborhood poverty were crucial determinants of racial disparities in terms of low birth weight in New York [71]. Finally, in his book *The color of law*: *A forgotten history of how our government segregated America*, Rothstein systematically elaborates on how federal, state, and local governments in the U.S. have created and reinforced racial segregation by introducing and implementing a series of regulations and policies [80].

### 5.2. Research Topic Identification: Co-Citation Cluster Analysis

**Figure 5:**
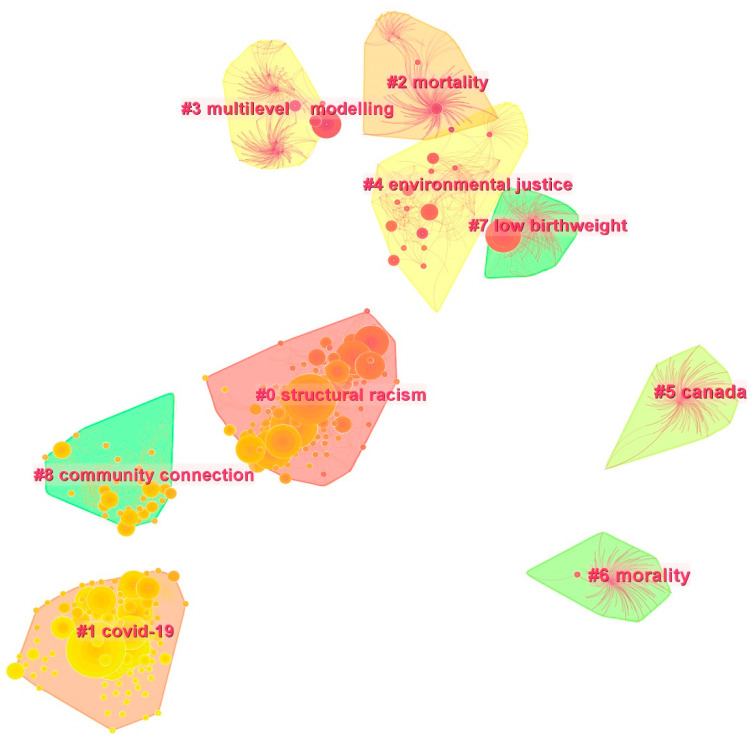
Clustering map of reference co-citations in RSH research.

The cited literature reflects key topics in the field. Thus, we identified document co-citation clusters based on a co-citation network to further understand the corresponding topics of representative literature in the knowledge domain of RSH. The cluster knowledge map outlines the network’s structural characteristics through the Modularity Q and Mean Silhouette scores. In general, Q > 0.3 indicates a significant cluster structure, while S > 0.5 implies that the cluster is reasonable [54]. In the generated network, the Modularity Q score was 0.8609, and the Mean Silhouette score was 0.9422, signaling that the cluster structure is significant and convincing. The network generated 25 co-citation clusters, from which we extracted the top 10 (Figure 5). The quantity of literature in each topic cluster varies greatly, with the largest cluster being #0 (structural racism), which contains 170 references, and the smallest cluster being #9 (ethnic density), which contains 58 references. In terms of temporal distribution, most of the topic clusters appear in the period of 2000–2005, suggesting that the time before 2005 was the initial stage of RSH research, which is consistent with the analysis of annual publication volume. The five largest clusters are as follows:

#0–Structural racism: Structural racism is a key topic in RSH research. Racism can help to explain health inequalities and poor health outcomes among minorities [48,81,82]. In addition, racism is often interwoven with place, creating inequality by differentially distributing opportunities and social resources to different racial groups [16,78]. Racism exposes minority groups to poor social conditions and risks such as unemployment, adverse environmental exposure, and resource shortages, which can harm health [83,84,85]. A total of 170 references were included in this cluster. The most cited articles are by Kramer and Hogue [45], Grady et al. [71], White and Borrell [49], and Subramanian et al. [76], all of which have been cited more than 30 times. They are among the top 10 most highly cited sources described in Section 5.1.

#1 COVID-19: The COVID-19 outbreak resulted in a disproportionate number of diagnoses and deaths among racial minorities, who are extremely vulnerable to COVID-19 due to the long-standing structural disadvantages of racism and racial residential segregation. Communities with greater proportions of Blacks or Hispanics and poverty tend to have higher COVID-19 diagnoses and mortality rates [86,87,88]. Communities with high percentages of minorities also lack vaccine distribution points [89]. Crowded housing and air pollution caused by segregation accelerated the spread of the virus and increased mortality rates [86]. Moreover, COVID-19 has perpetuated deeply rooted racial health inequalities and exposed vulnerable populations to increased security threats. There are 126 articles included in this cluster. Bailey et al. [78] ranked first with 72 citations followed by Rothstein [80] and Williams et al. [48]. They are among the top 10 highly cited sources described in Section 5.1.

#2 Mortality: Disease mortality has always been a vital component of health outcomes. In 1998, Bird and Bauman and Guest et al. highlighted the crucial effects of residential segregation and socio-economic structure on infant mortality [90,91]. With the expansion of research, more comprehensive studies have been conducted on the mortality rates associated with different illnesses such as CVD [92] and breast cancer [93,94,95]. There are 26 references in this cluster. The most cited source, Acevedo-Garcia [96], was cited eight times followed by Williams and Collins [13], then Pickett and Pearl [97]. Acevedo-Garcia built a conceptual framework to explain the link between residential segregation and health from an epidemiological angle, using tuberculosis as an example, while underlining the application of two segregation measures: the exposure index and the segregation index [96]. By reviewing the literature, Williams and Collins found that structural racism promoted the formation of racial residential segregation and played a significant role in racial health inequality [13]. Pickett and Pearl reviewed 25 studies involving neighborhood socio-economic background and health outcomes; they found that 23 studies indicated that neighborhood socio-economic context was significantly correlated with health outcomes when controlling for individual socio-economic status [97].

#3 Multilevel modelling: Multilevel statistical analysis is a common method in RSH research [76,98,99,100]; it encompasses spatial error models, linear and Poisson regression models, multinomial logistic regression, hierarchical and multilevel models, and ordinary least squares (OLS) regression. The studies explored the link between residential segregation and specific health outcomes by building a multilevel model that can accurately analyze impacts on health at different levels (e.g., the individual and community levels). This cluster contains 113 articles. The top three most cited sources are Williams and Collins [13], Pickett and Pearl [95], and Jackson et al. [101]. The study by Jackson et al. on mortality in the U.S. implies that that residential segregation is associated with racial disparities in mortality [101].

#4 Environmental justice: Environmental justice has become a vital topic, indicating that residential segregation produces environmental injustice to a certain extent. Minority and low-income groups often face greater environmental hazards [23,102,103], as well as a spatially unequal distribution of the built environment [104,105,106,107,108,109]. The three most cited sources are Morland et al. [66], Zenk et al. [110], and Schulz et al. [111]. Morland et al. and Zenk et al. highlighted the distribution of supermarkets across racial communities, showing that the number of supermarkets in the poorest and Black communities is much lower than in wealthy and White communities [66,110], and poor communities are more likely to consume alcoholic beverages [66]. Poor food environments prevent minorities from consuming the nutrients they need for good health. Schulz et al. analyzed the socio-economic inequalities between Blacks and Whites and disparities in access to health-related material resources, and argued that this plays a crucial role in unequal health outcomes [111].

#5 Canada: Canada, as a country with a high degree of immigration, is often used in RSH research to study immigrants’ spatial assimilation and ethnic residential segregation [112,113,114].

#6 Morality: Morality emphasizes the majority’s sense of morality, such as class prejudice and cultural preferences for the integration or segregation of minorities [115]. Pow and Kovacheff et al. showed that negative moral order has the capacity to foster class exclusion and segregation, which is likely to cause social polarization and foster segregation [116,117].

#7 Low birth weight: This area focuses on the racial disparities and influencing mechanisms of low birth weight among infants caused by residential segregation [71,118,119].

#8 Community connections: This area involves the interactions and community networks among the members of a community. Gibbons and Yang found that Blacks have weaker community connections in White or integrated communities. In contrast, living in a racial minority community can facilitate the formation of strong social networks [120].

#9 Ethnic density. Ethnic density refers to the impact of concentrations of one race in a particular geographic area or different degrees of ethnic density on health outcomes; it is often measured in geographic units. A study on ethnically dense areas and the risk of pre-term birth across seven races showed that higher ethnic density increased this risk among Black women, decreased the risk among White women, and was not associated with (or had a weak protective effect against) pre-term birth among Hispanics and Asians [121].

## 6. Research Hotspots and Research Frontiers

### 6.1. Research Hotspots: Keyword Cluster Analysis

**Figure 6:**
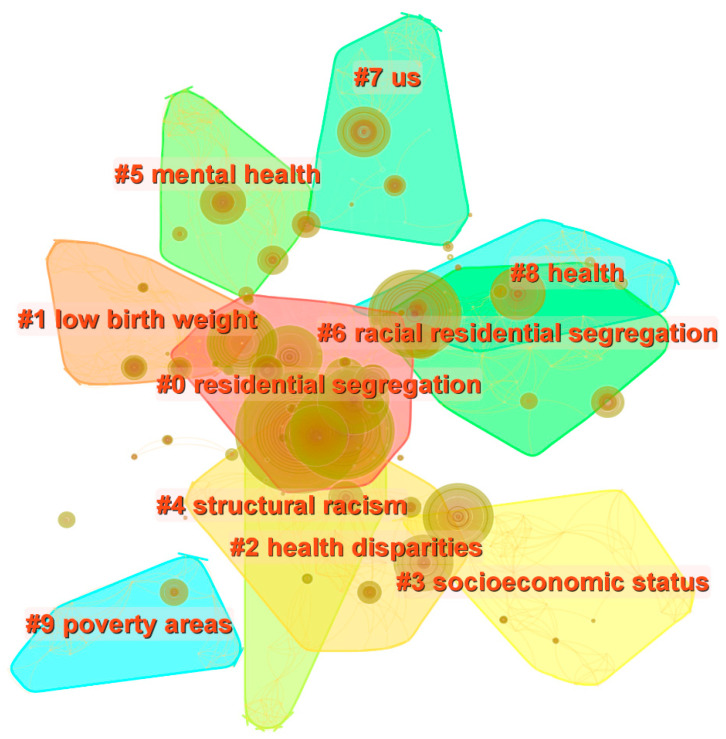
Clustering map of keyword co-occurrences in RSH research.

Keywords reflect an article’s core content and ideas. As such, the high frequency of keyword co-occurrences indicates research hotspots in a given subject area. By using CiteSpace, we derived the top 10 keywords (in order of highest frequency): residential segregation, United States, health, disparity, socio-economic status, African American, mortality, health disparity, racial disparity, and race. Next, we performed clustering and formed a map of keyword co-occurrences found in RSH research (Figure 6). The Modularity Q score was 0.7614, and the Mean Silhouette score was 0.9084, indicating that the cluster structure is significant and reasonable. Combined with the keyword cluster map, we divided the hotspots into the following four types of research: (1) studies on health inequalities between racial minorities and racial majorities, with the keyword cluster residential segregation, health disparity, and racial residential segregation; (2) empirical research with the United States and poverty areas as the chief research area with the keyword cluster United States (US) and poverty area; (3) studies on the mechanistic pathways of influence of residential segregation leading to adverse health outcomes with the keyword cluster structural racism and socio-economic status; and (4) research on specific adverse health outcomes caused by residential segregation with the keyword cluster mental health and low birth weight.

### 6.2. Research Frontiers: Burst Keyword Analysis

**Figure 7:**
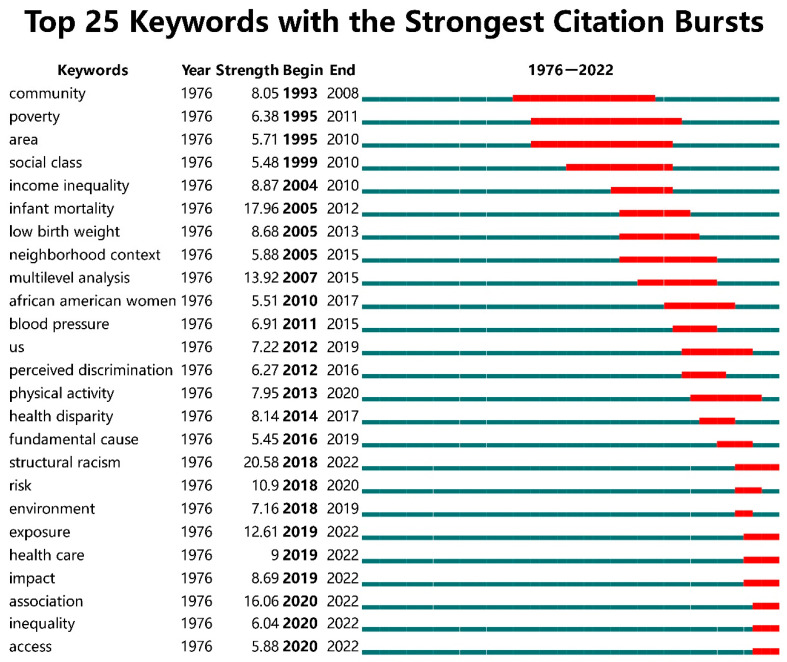
Top 25 keywords with the strongest citation bursts of RSH research from 1976 to 2022.

The exploration of research frontiers helps us to forecast future research trends. We achieve algorithm analysis and visualization using Kleinberg’s burst detection algorithm [122] and CiteSpace software. CiteSpace provides the function of burst detection, which can delineate a certain discipline’s research frontier and development trend. In the keyword burst analysis mapping, the blue line represents the entire research period, and the red line denotes the duration of citation bursts. Thus, bursts contain two directions: strength and duration. If the duration is longer, the burst strength value is greater, suggesting that the topic is influential at a certain stage. If the keyword has continued since its appearance in a certain year, it can be considered a continuous hotspot and research frontier. Figure 7 depicts the top 25 keywords with the strongest citation bursts in RSH research over different periods. The figure indicates that all keywords have a strength value above 5 with the highest at 20.58 (structural racism) and the lowest at 5.45 (fundamental cause). Poverty was the keyword with the longest duration (17 years). The development of the field can be divided into four stages, suggesting a significant shift from focusing on community, poverty, infant mortality, and social class to residential environmental exposure, structural racism, and health care.

(1) 1976–2003: This period laid the foundation for RSH research. The burst strength value of community, poverty, area, and social class were high and lasted for more than 10 years, which indicates that socio-economic background differences and social class differences were the first subjects to receive attention, comprising a pioneering area in the development of RSH research.

(2) 2004–2011: The topics of RSH research became diversified in this period. The top three keywords for burst strength values were infant mortality, multilevel analysis, and low birth weight, which means that research progressed in terms of methodology and content. The effects of residential segregation on specific health outcomes (e.g., infant mortality, low birth weight, high blood pressure) became the focus of attention. The multilevel approach may better detect the effects of metropolitan, community, and individual-level factors on health outcomes [4]. This method became the dominant approach for empirical studies in this stage.

(3) 2012–2017: As the impact of segregation on adverse health outcomes deepened, causality studies were strengthened. Scholars were no longer limited to debating whether segregation causes adverse health outcomes, but further explored the mechanisms and pathways of external influences. Thus, the mediators of segregation and health, such as perceived discrimination and physical activity, became the chief themes during this period [123,124,125].

(4) 2018–2022: In 2018, except for risk and environment, which were maintained for a short time, the burst duration of keywords (including structural racism, exposure, health care, impact, association, inequality, and access) continued, leading to new research trends. The burst duration of structural racism, exposure, and association is over 12. The research frontiers are summarized as follows: (1) the relationship between structural racism and health inequalities [126,127,128]; (2) the unique relationship between structural racism and place-based physical environments, and between structural racism and health care accessibility [16,129,130]; and (3) the effects of long-term exposure to segregated environments [131,132]. These studies suggest that inadequate access to socio-economic and health care resources resulting from structural racism disproportionately exposes minorities to a poor health environment.

## 7. Discussion

Using CiteSpace, we conducted a comprehensive bibliometric analysis of developments in RSH research. Residential segregation has become an important topic in the context of health and has produced abundant achievements.

An interesting finding in the analysis of collaborative networks is that the representative authors and institutions are mostly affiliated with each other. For example, Thorpe and Gaskin are both at the Johns Hopkins School of Public Health, Williams is at Harvard University, and LaVeist previously worked at the University of Michigan and the Johns Hopkins University School of Public Health. Thus, we can assume that productive authors make institutional contributions to RSH research. Universities are likely to invest more financial resources and resources to help scholars carry out their research, which corroborates the finding that the top 10 institutions are all universities. Research produced in the U.S., the country with the most abundant research results, is closely related to its domestic racial residential segregation, which is due to its historical-political background and immigration [133,134].

Review articles comprise the majority of the top 10 MCPs, indicating that the summary, based on a large number of past studies, can help scholars to better understand the development of this field. The highly cited literature underlines the relationship between racism and health inequalities and hints at future research directions. In addition, we focused on the top five theme clusters and interpreted the included highly cited literature. The top three clusters are structural racism, COVID-19, and mortality. First, structural racism was the keyword with the highest strength value, which is consistent with the results of the co-citation cluster analysis. A constant force of influence is evident in the impact of social structural disadvantages for health. At the same time, we also noted the changes taking place in the world including shifts in attitudes towards Blacks (“Black Lives Matter”) and other ethnic groups as well as the implementation of many anti-discriminatory and inclusion policies, which have been changing the situation [135].

Second, the prevalence of COVID-19 has led to rapid, widespread interest in a short period. COVID-19 contributes to the disproportionate exposure of minorities and other vulnerable groups to health threats. Disparities in health-related resources and socioeconomic background lead to racial disparities in diagnosed cases and mortality. Some agencies have identified this situation and responded with policy changes, for example, by enhancing diagnosis and support for underserved populations and prioritizing vulnerable groups for vaccination [136,137].

Third, the prominence of mortality reflects the significant threat of residential segregation. In existing studies, factors such as environmental pollution, lack of access to health care when living in remote areas, and socioeconomic factors are important contributors to high mortality rates [138,139]. In response to this problem, policies (e.g., social mixing in housing policies, environmental justice policies, and policies to improve the coverage and quality of health care services) have attempted to address issues such as unbalanced economic and resource distribution and environmental exposure, somewhat reducing mortality disparities.

Based on our bibliometric analysis of the field and reading of representative literature, we not only formed an overview of the development of RSH research, but also identified limitations.

(1) Most of the current findings and causal relationships on the health effects of residential segregation are empirical studies conducted in the U.S. As a global social phenomenon, residential segregation may lead to different empirical outcomes regarding the health effects of minority groups in countries or cities with different social backgrounds, ethnic compositions, and levels of social development. However, there are few empirical comparisons and comprehensive studies that target minority–majority discrepancies between different social contexts and countries. Hence, a comparative study of different regions or backgrounds may provide useful ideas and strategies for addressing racial health inequalities.

(2) Existing research has mostly focused on the effects of segregation between racial minority and majority groups, such as the effects of segregation between Blacks and Whites and between Hispanics, Asians, and Whites. It is not yet entirely clear whether the adverse health effects of residential segregation are consistent across social classes in the same racial group, such as the health effects of residential segregation between lower and higher income groups in the same racial group.

(3) In studies on causality, most scholars have used multilevel model analyses to test for the effects of residential segregation on health outcomes. As a common method, multilevel model analyses have produced rich findings for RSH research, but the limitation is that studies have examined the impact of residential segregation on health through sample data for a specific period of time and lack long-term dynamic monitoring of residents’ health status. Studies have not been able to discern whether the impact of segregation on health occurs at each stage of life and whether the impact is consistent in each phase.

## 8. Conclusions

The relationship between residential segregation and health is diverse and complex, involving sociology, economics, epidemiology, political science, and other disciplines. In the context of the development of healthy cities, research in this area is of great significance. The results will help professional scholars to understand the discipline’s evolution more comprehensively. Based on the WOS core database and CiteSpace visualization software, we conducted a series of analyses of RSH research, presenting an overview and development trends in this field. We answered all the research questions.

First, there has been a general upward trend in the number of RSH studies published from 1976 to 2022. In particular, the number of publications accelerated significantly after 2014, indicating a continued rise in research interest. Relevant journals cover categories such as medicine, public health, racial health, and epidemiology.

Second, Thorpe, LaVeist, Williams, and Gaskin are the most representative scholars, and there is a strong collaborative network relationship between the first three authors. The U.S. is the leading country in this field with significant academic influence, followed by England and Canada. Among the 384 institutions involved, the University of Michigan has made the largest contribution, followed by Columbia University and Harvard University.

Third, in terms of landmark literature and hot topics, the 10 most highly cited sources include Bailey et al. [78], Rothstein [80], and Kramer and Hogue [45]. We identified 10 major clusters, with structural racism, COVID-19, mortality, multilevel modelling, and environmental justice representing the most popular topics in RSH research. Research hotspots included disparity, disorder, United States, African American, physical activity, area, mental health, blood pressure, low birth weight, residential segregation, and socio-economic status. The research frontier of RSH has shifted significantly from focusing on community, poverty, infant mortality, and social class to residential environmental exposure, structural racism, and health care.

This study is not without limitations. We chose to derive data from the core database of WOS, and we only selected English-language articles. Although our results suggest that North American and European countries are the largest contributors to RSH research, we did not cover articles involving other national languages; doing so would require a broader literature search and data analysis. However, we believe that visual analysis grounded in a large amount of literature-based data will contribute to this area and bring more attention to health issues. Finally, it is necessary to provide a more comprehensive and precise understanding of RSH research by combining the results of the quantitative data analysis with the reading of literature content to help researchers better grasp the topics, developments, and frontiers in the field.

## 9. Future Research

With the spread of COVID-19 and its subsequent health effects, health issues have inevitably become an important challenge for people. Based on the bibliometric analysis and a review of relevant literature, further research in the field of RSH should be explored in depth in terms of the following aspects:

(1) The relationship between structural racism and health, as well as its mediators, and the effects of community environmental exposure on health and other mechanisms beyond racism (e.g., stress, nutrition, crime, housing, and transportation) represent paths for future research. Specifically, investigation of internal mechanisms should go beyond the mere demonstration of adverse health outcomes and become a future development trend, which would benefit scholars in proposing targeted interventions.

(2) Although there are certain barriers in conducting cross-country comparative research, such as lack of project funding and differences in cross-country cultural backgrounds, it is necessary to strengthen research on the health effects of residential segregation on minority groups in different socio-economic and cultural contexts, and to conduct comparative studies in various countries and regions, which may on the one hand provide insight into measures and policies of health equity, while on the other hand, it can promote the synthesis of theories and exploration into the possibility of forming a grand theory. Therefore, it is even more necessary to strengthen cooperation and academic exchanges among international scholars and to find points of interest for mutual cooperation.

(3) It is necessary to expand research on RSH between different social classes within and outside of the same race. In terms of methodology, longitudinal studies rooted in the dimension of time should be conducted to observe individuals’ spatial/temporal characteristics and to clarify the causal link between diverse elements and health.

## Figures and Tables

**Table 1:** Top 10 journals ranked by the number of publications in RSH research.

No.	Quantity	Proportion	Journal Names
1	97	7.50	Social Science & Medicine
2	51	3.94	Journal of Urban Health–Bulletin of The New York Academy of Medicine
3	50	3.86	Health & Place
4	45	3.48	International Journal of Environmental Research and Public Health
5	43	3.32	American Journal of Public Health
6	26	2.01	Journal of Racial and Ethnic Health Disparities
7	24	1.85	PLOS ONE
8	21	1.62	Ethnicity & Disease
9	20	1.55	American Journal of Epidemiology
10	19	1.47	Annals of Epidemiology

**Table 2:** Top 10 productive authors in RSH research.

No.	Author	Quantity	No.	Author	Quantity
1	Thorpe, R. J.	38	6	Osypuk, T. L.	14
2	LaVeist, T. A.	29	7	Kramer, M. R.	14
3	Williams, D. R.	20	8	Krieger, N.	14
4	Gaskin, D. J.	19	9	Gibbons, J.	12
5	Kershaw, K. N.	16	10	Bowie, J. V.	12

**Table 3:** Top 10 research institutions in the field of RSH.

No.	Quantity	Proportion	Institution	Year
1	95	7.34	University of Michigan	1999
2	68	5.26	Columbia University	2003
3	59	4.56	Harvard University	1999
4	55	4.25	Johns Hopkins Bloomberg School of Public Health	2004
5	51	3.94	University of North Carolina	2003
6	47	3.63	University of California- San Francisco	2006
7	40	3.10	Emory University	2008
8	40	3.10	Northwestern University	2005
9	40	3.10	University of Washington	1998
10	35	2.70	University of California, Berkeley	1999

**Table 4:** Top 10 most highly cited references in RSH research.

Frequency	Author	Title	Source	Year	Centrality
66	Bailey et al. [78]	Structural racism and health inequities in the USA: Evidence and interventions	Lancet	2017	0.04
51	Rothstein [80]	The color of law: A forgotten history of how our government segregated America	Liveright	2017	0.04
44	Kramer and Hogue [45]	Is segregation bad for your health?	Epidemiologic Reviews	2009	0.05
38	Grady et al. [71]	Racial disparities in low birth weight and the contribution of residential segregation: A multilevel analysis	Social Science & Medicine	2006	0.04
37	White and Borrell [49]	Racial/ethnic residential segregation: Framing the context of health risk and health disparities	Health & Place	2011	0.04
36	Williams and Collins [13]	Racial residential segregation: A fundamental cause of racial disparities in health	Public Health Reports	2001	0.03
35	Williams et al. [48]	Racism and health: Evidence and needed research	Annual Review of Public Health	2019	0.00
34	Acevedo-Garcia et al. [4]	Future directions in residential segregation and health research: A multilevel approach	American Journal of Public Health	2003	0.27
33	White et al. [79]	Elucidating the role of place in health care disparities: The example of racial/ethnic residential segregation	Health Services Research	2012	0.09
33	Subramanian et al. [76]	Racial residential segregation and geographic heterogeneity in Black/White disparity in poor self-rated health in the U.S.: A multilevel statistical analysis	Social Science & Medicine	2005	0.04

## Data Availability

Not applicable.
